# Reimagining the Research Approach to Tuberculosis†

**DOI:** 10.4269/ajtmh.17-0999

**Published:** 2018-01-22

**Authors:** Anthony S. Fauci, Robert W. Eisinger

**Affiliations:** Office of the Director, National Institute of Allergy and Infectious Diseases, National Institutes of Health, Bethesda, Maryland

## Abstract

Controlling and ultimately ending tuberculosis (TB) as a public health scourge will require a multifaceted and comprehensive approach involving the intensification of public health efforts, including scaling-up the delivery of current diagnostic, preventive, and therapeutic tools. However, a critically important element in the effort to end TB is an accelerated biomedical research effort to address the many unanswered questions about the disease process itself and to develop improved and innovative countermeasures. An intensive effort toward these research goals will facilitate the achievement of the aspirational goal of ending TB.

Tuberculosis (TB) is a disease of historical importance, accounting for at least 1 billion deaths over the past two centuries, more than the combined number of deaths from malaria, smallpox, human immunodeficiency virus (HIV)/acquired immune deficiency syndrome (AIDS), cholera, plague, and influenza.^1^ More than 2 billion people are estimated to be latently infected with *Mycobacterium tuberculosis* (*Mtb*), and TB is the leading cause of death among infectious diseases and one of the top 10 causes of death worldwide. In 2016, an estimated 1.7 million TB deaths occurred. There were an estimated 10.4 million new TB cases in 2016, 10% of which were among individuals living with HIV infection. About 490,000 of the new cases were multidrug-resistant TB (MDR-TB), with 6.2% of those cases identified as extensively drug-resistant TB (XDR-TB).^2^

The first World Health Organization (WHO) Global Ministerial Conference on Ending TB in the Sustainable Development Era: A Multisectoral Response (Moscow, November 16–17, 2017) was an historical event that brought attention to the compelling need to reassess the global effort to end this historic scourge. The conference highlighted the importance of implementing effectively the tools that we already have for preventing TB and diagnosing and treating/caring for people infected with *Mtb*, and it underscored the need for additional, improved tools for this purpose, with the latter resulting from biomedical research.^3^ At the conference, one of us (A.S.F.) outlined how we might “reimagine” our research response to TB and bring TB research into the twenty first century with the application of new diagnostic, therapeutic, and vaccine platforms.

The increasing incidence of MDR-TB and XDR-TB during the past few years has sparked heightened attention to the urgent need for a comprehensive “tool kit” of new and better strategies to prevent, diagnose, treat, and control TB and drug-resistant TB.^4^ We must not take just an incremental approach to another drug or another diagnostic. TB is an ancient disease; however, we need to understand it in modern terms and use cutting-edge technologies to ask and answer questions that were never addressed in the first place because years ago, we felt that we had effective drugs that could cure the disease. It is true that elegant research has been conducted in the field of TB over the past few years; however, because of the complexity of this disease, critical questions remain unanswered regarding the pathogenesis of TB. In addition, there remains a paucity of innovative and highly effective interventions. We must change our mind-set about our end game in the approach to TB research. Our goal should be to transform the entire field.

The current situation with TB research contrasts dramatically with the unprecedented advances in HIV/AIDS research made in the > 36 years since HIV was first reported.^5,6^ We now have a robust HIV/AIDS tool kit that includes techniques to detect as few as one to two copies of HIV RNA in the blood with a simple and widely available test^7^; more than 30 FDA-approved antiretroviral drugs, which when used in combination, result in a substantial projected life-expectancy and a return to normal daily activities in the vast majority of treated individuals^8^; and safe and highly effective prevention strategies including pre-exposure prophylaxis to prevent acquisition of HIV.^9^ In addition, several promising vaccine candidates are presently being tested in large-scale, international Phase 2 clinical trials.^10–12^ We should not settle for any less when it comes to TB.

Recently, efforts have been made to emulate the HIV research model in the fight against TB. In this regard, the U.S. Government’s Global Tuberculosis Strategy is closely aligned with the WHO’s End TB Strategy, “A World Free of TB.” Both place a high priority on intensified research and innovation to successfully achieve an end to TB.^13,14^ Various aspects of the TB research agenda involving pathogenesis, diagnostics, therapeutics, and vaccines were presented by A.S.F. at the Moscow conference.

## PATHOGENESIS RESEARCH

More than 130 years after the discovery of *Mtb* as the etiologic agent of TB, we still know surprisingly little about the precise mechanisms of TB disease pathogenesis. Generations of research advances and technologies applied to other diseases have bypassed the field of TB research. Key questions remain unanswered, particularly regarding the complex interactions between the pathogen and the host. This gap in our knowledge will benefit greatly from interdisciplinary, systems biology approaches to TB pathogenesis that include computational and mathematical modeling of the complex biological interactions between pathogen and host. Such efforts will contribute greatly to enlightening the still opaque areas of maintenance of latency, escape from latency, disease activation, and correlates of immunity.^15^ In addition, key research challenges in pathogenesis must be addressed with regard to TB/HIV co-infection, including the factors associated with higher rates of progression to active TB and the accelerated course of HIV in coinfected individuals; different clinical/radiographic manifestations of pulmonary TB; and reduced immune control and a greater degree of extrapulmonary TB dissemination.^16^ Limitations of animal models of dual infection are also an impediment that must be addressed.

## DIAGNOSTICS

The GeneXpert MTB/rifampicin resistant diagnostic (Cepheid, Sunnyvale, CA) has been a welcome addition to the TB diagnostic armamentarium.^2^ However, there is still a need for improved and transformative TB diagnostics to overcome the severe limitations of antiquated, nonstandardized, and imprecise techniques which are used presently in most settings. In addition, the application of twenty first century diagnostic technologies that can detect *Mtb* in a variety of clinical specimens from multiple body sites in addition to sputum, as well as advanced approaches for monitoring and predicting treatment outcomes are a priority. If we can detect a single copy of HIV RNA in the blood of an HIV-infected individual and track disease progression with viral load testing, we should be able to develop a comparable diagnostic and disease monitoring capability for TB, as far-fetched as this might seem at present.

The diagnosis of TB in coinfected individuals is complicated because extrapulmonary and paucibacillary pulmonary disease is more common with HIV infection, and these forms of the disease require greater test sensitivity. Yet, TB diagnosis is still focused predominantly on sputum specimens, which can be difficult to obtain in HIV-infected people, particularly children.^17,18^ For all TB patients, there is a critical need for rapid, inexpensive, and accurate “point-of-care” molecular diagnostics that can rapidly differentiate between drug-sensitive and drug-resistant forms of *Mtb*, such that appropriate drug regimens can be prescribed, based on the molecular profile of the pathogen. Although an extensive data bank of genomic sequences from numerous *Mtb* strains is available, additional basic research is needed on gene mutations resulting in drug resistance to help inform treatment decisions.^19^

## THERAPEUTICS

World Health Organization’s recent report *Antibacterial Agents in Clinical Development: An Analysis of the Antibacterial Clinical Development Pipeline, including Tuberculosis* noted the critical need for additional support for basic science on *Mtb*, drug discovery, and clinical development of better TB treatment strategies.^20^ Current treatment regimens recommended by WHO include up to four drugs for 6 months, but result in an insufficient ∼83% cure rate globally in newly diagnosed individuals. World Health Organization also has cited the desperate need for newer, non-toxic drugs and shorter regimens for treatment of MDR-TB because the current regimens include four to seven drugs for 9–20 months, with only a ∼54% cure rate globally.^2^

Approximately half of MDR-TB patients have resistance to second-line drugs, and XDR-TB treatment is effective in only about 30% of these individuals.^2^ Despite the urgent need for better treatments, only a few new drugs and lead agents are in the drug pipeline.^2,21^ There remain numerous hurdles that must be overcome in the successful development of safe and effective TB therapeutics, including the length and complexity of treatment regimens, challenges to adherence, toxic side effects, drug–drug interactions, and drug resistance. There also are unique challenges for TB treatment in HIV-coinfected individuals, including drug–drug interactions between TB therapy and antiretroviral therapy, which can result in additive toxicities, risk of immune reconstitution inflammatory syndrome, and the limited availability of pediatric drug formulations.^22^

One initiative to address these challenges is the TB drug accelerator, an innovative international collaboration between seven pharmaceutical companies and six research institutions. The project is designed to address the shortage of new TB drugs by funding early-stage TB drug discovery.^23^ In an effort to boost clinical research on TB, the National Institutes of Health (NIH) has used the infrastructure and capacity of its extensive HIV clinical trials networks to conduct critical studies of potential TB treatment regimens, including a 1-month short-course of rifapentine/isoniazid to prevent active TB in HIV-infected patients with latent TB. These networks have also conducted MDR-TB treatment studies of bedaquiline and delamanid, alone and in combination, as well as studies of optimized and individualized MDR-TB therapy for HIV-infected and HIV-uninfected children.^24^

We should not despair of the possibility of achieving the goal of a combination of drugs directed at multiple targets administered for a substantially shorter period of time than is presently required and that will cure an individual infected with any strain of *Mtb*. We never would have imagined 25 years ago that we could durably suppress HIV replication to undetectable levels with three antiretroviral drugs given as a single pill once per day. Our goals for the ultimate therapy of TB should be no less aspirational.

## VACCINES

A safe and effective TB vaccine is urgently needed to protect against all forms of TB in adults and adolescents.^25,26^ Recent modeling exercises underscore that a new TB-preventive vaccine that is 60% efficacious and provided to 20% of adults and adolescents globally could avert ∼60–70 million cases in its first 25 years of use, and an infant vaccine could potentially avert 6–7 million new TB cases.^26^

However, it appears that we are far from our goal of a useful TB vaccine. The BCG vaccine is not effective in preventing adult pulmonary TB, which is the major transmissible form of the disease, and the vaccine is no longer recommended for HIV-coinfected children.^27^ With regard to the development of new vaccine platforms to prevent TB, there remain many challenges. These include unanswered questions about the pathogenesis of TB infection and disease as mentioned previously; gaps in our knowledge of the precise nature of protective immunity; a lack of reliable and sensitive correlates of immune protection; limited understanding of the precise nature and effectiveness of pulmonary host defenses to *Mtb*, whether innate and/or adaptive; and limited predictive value of effectiveness in animal models.^28^ Clearly, the development of a safe and effective vaccine remains one of the most formidable TB biomedical research challenges, but a challenge that we must vigorously undertake.

In summary, we cannot adequately address the broad issue of infectious diseases in global health without directly addressing TB, the greatest infectious killer. Now is the time to reinvigorate our response to TB by building on the momentum of the successful November 2017 Ministerial Conference in Moscow. It is critical to reimagine what is possible with innovative biomedical research and to accelerate our multifaceted research programs with increased and sustained resources, including critical support for training the cadre of the next generation of TB researchers. With robust, creative, and aggressive research efforts and by rapidly translating research results into TB control strategies that can be implemented globally, we can reach the aspirational goal of ending TB.
